# Endothelial cell specific molecule 1 is today a relevant marker of respiratory failure in sepsis and polytrauma patients

**DOI:** 10.1186/cc11797

**Published:** 2012-11-14

**Authors:** P Lassalle, N De Freitas Caires, L Portier, A Palud, E Parmentier, J Pastre, C Shah, A Scherpereel, D Mathieu, M Delehedde

**Affiliations:** 1 Lunginnov, R&D Departement, Lille, France; 2 ICU, Calmette Hospital, Lille, France; 3 University, Philadelphia, PA, USA; 4 Service de Pneumologie, Calmette Hospital, Lille, France

## Background

Initially described in endothelial cells from the lung, endothelial cell specific molecule 1 (ESM-1), also called endocan, is a circulating proteoglycan of 50 kDa that can be easily quantified in biological fluids (sera, plasma). This robust macromolecule is upregulated in the presence of proinflammatory agents and has been described as a biomarker of endothelial dysfunction in several disease conditions.

## Methods

We here compared and analyzed the results obtained from studies using a sandwich-type ELISA (DIYEK H1, Lunginnov) for evaluating whether ESM-1 could be useful to predict organ failure in ICU patients such as septic patients and polytrauma patients (*n *= 67). Data baseline characteristics, APACHE II score, procalcitonin (PCT) and C-reactive protein (CRP) levels and ICU course such as ICU length of stay were also evaluated for each ICU patient admitted and compared with ESM-1 levels.

## Results

In all studies we observed that elevated blood levels of ESM-1 correlated with the severity of sepsis and the poor outcome in patients with severe sepsis or in septic shock at ICU admission. We furthermore evaluated the presence of ESM-1 and ESM-1 degradation products in the biological fluids (sera, plasma and urines) from septic patients by ELISA, immunoprecipitation and western blotting procedures. When compared with data baseline characteristics, ESM-1 levels were shown to not correlate with CRP and PCT levels. Interestingly we may suggested from our analysis that blood ESM-1 >3 ng/ml represents an additional criterion of severity in a context of SIRS that may be useful in the ICU. At 72 hours, ESM-1 exhibited a clear predictive value for acute lung injury (sensitivity 85%; specificity 100%) in septic patients. In comparison, lower levels of serum ESM-1 in polytrauma patients were associated with development of acute lung injury and reflect respiratory failure.

## Conclusion

No circulating molecule was up to now described as an indicator of respiratory failure in septic patients and in polytrauma patients. In a context where respiratory failure is still the first cause of death in sepsis, our study analysis suggests that blood levels of ESM-1 may be a useful early biomarker of lung tissue injury and respiratory failure in ICU patients.

